# Exploring the Interplay between Sleep Quality, Stress, and Somatization among Teachers in the Post-COVID-19 Era

**DOI:** 10.3390/healthcare12151472

**Published:** 2024-07-24

**Authors:** Stefania Mancone, Stefano Corrado, Beatrice Tosti, Giuseppe Spica, Francesco Di Siena, Pierluigi Diotaiuti

**Affiliations:** Department of Human Sciences, Society and Health, University of Cassino and Southern Lazio, 03043 Cassino, Italy; s.mancone@unicas.it (S.M.); stefano.corrado@unicas.it (S.C.); beatrice.tosti@unicas.it (B.T.); giuseppe.spica@unicas.it (G.S.); francesco.disiena@unicas.it (F.D.S.)

**Keywords:** teacher well-being, psychological stress, sleep quality, somatization, post-COVID-19 challenges, educational health policy

## Abstract

(1) Background. The post-COVID-19 era has imposed unique challenges on educators, significantly impacting their psychological and physical well-being. This study examines the interrelationships among psychological stress, sleep quality, and somatization in a sample of teachers, elucidating the impact of these factors during the ongoing recovery from the pandemic. (2) Methods. Using validated instruments such as the Pittsburgh Sleep Quality Index (PSQI) and the Mesure du Stress Psychologique (MSP), this research investigates how stress and sleep disturbances correlate with somatization among teachers. The study also considers the influence of demographic factors such as age, gender, and years of experience. (3) Results. The results indicated that sleep quality significantly correlates with both psychological stress and somatic pain, emphasizing the crucial role of sleep in managing stress-induced physical symptoms. Additionally, the fear of COVID-19 significantly exacerbates these effects, illustrating the complex interplay of psychological and physical health factors during the pandemic. Contrary to initial hypotheses, demographic factors such as gender, age, and years of experience did not significantly influence these primary relationships. (4) Conclusions. The findings emphasize the necessity of addressing both psychological stress and sleep quality to mitigate their combined effects on somatization. Educational institutions and policymakers are urged to develop targeted interventions that address these issues to support teachers’ health and well-being in a post-pandemic landscape.

## 1. Introduction

Stress is a prevalent issue that can have significant impacts on individuals, affecting various aspects of their health and well-being. One area where stress can manifest is in sleep disturbances, which have been linked to a range of physical symptoms [1,2]. Teachers often experience muscular pain, sleep disturbances, headaches, and gastrointestinal problems, which are not isolated from psychological effects like irritability and depression [3,4,5]. These compounded burdens can significantly affect teachers’ productivity and effectiveness, leading to a cycle of stress and poor health that may result in burnout [6,7,8].

De Sousa et al. [9] explored occupational stress and sleep quality in teachers, revealing an association between poor sleep quality and physical and emotional symptoms. Fontana et al. [10] investigated the relationship between physical activity, sleep quality, and stress among teachers during the COVID-19 pandemic. The study showed that poor sleep exacerbated the high levels of stress reported by teachers, with a significant portion experiencing poor sleep quality. During challenging times like the COVID-19 pandemic, the combined effect of perceived stress and poor sleep has been found to account for a substantial portion of physical health symptoms, underscoring the intricate relationship between stress, sleep, and physical well-being [11]. Sleep disturbances have been identified as significant symptoms in various mental health conditions, including depression, anxiety, and PTSD, indicating the interconnected nature of mental health, stress, and sleep quality [12]. Studies have also stressed the bidirectional relationship between physical symptoms and sleep disturbances, particularly in populations facing specific health challenges [13,14,15].

Cropley et al. [16] examined job strain, work rumination, and sleep in school teachers, finding that teachers with high job strain reported poorer sleep compared to the general population. This suggests that work-related factors significantly influence the sleep quality of teachers. Gluschkoff et al. [17] studied stressful psychosocial work environments and depressive symptoms among primary school teachers, showing that teachers not only experience higher levels of work-related stress but also exhibit more symptoms of poor mental health and sleep deprivation compared to other professions.

The COVID-19 pandemic has dramatically reshaped educational norms and expectations, magnifying the stressors faced by teachers. The sudden shift to remote and hybrid teaching models required teachers to adapt quickly to new formats and technologies, often without adequate preparation or support [18,19,20,21]. This transition introduced new challenges in maintaining student engagement and managing classroom dynamics in virtual settings [22,23,24]. The blurring of work-life boundaries and the constant need to balance personal well-being with professional responsibilities have further heightened stress levels among educators [25,26,27,28,29,30].

Research by Lizana et al. [25] highlighted the significant impact of the COVID-19 pandemic on teachers’ quality of life, suggesting that teleworking during the health crisis could have contributed to psychosocial health issues and physical burnout due to stress and work exhaustion. Studies such as those by Kotowski et al. [31] and Ouellette et al. [32] have emphasized the long-term effects of stress and burnout on teachers, linking these conditions to lower self-efficacy, reduced job satisfaction, and poor physical and psychological health.

The chronic exposure to stress reported by Khalifa et al. [33] has been associated with various stress-related physical symptoms such as tiredness, headaches, gastrointestinal disturbances, chest pain, and back pain among teachers. This can indicate a direct relationship between occupational stress and the manifestation of physical health issues in educators.

Recent studies such as those by Almansour [34], Gao [35] and Ferguson et al. [36] have also shown a direct correlation between stress and physical symptoms among teachers, with findings indicating a notable association between teachers’ back pain and perceived stress. Ratanasiripong et al. [37] highlighted the strong positive correlations between emotional exhaustion, depersonalization, and various physical symptoms like fatigue, cognitive weariness, and stress among teachers.

In addition, studies by Wang et al. [38] and Fontana et al. [10] have shown that sleep quality acts as a crucial mediator in the relationship between stress and depressive symptoms, indicating that addressing sleep quality could potentially alleviate the impact of stress on mental health outcomes.

Studies have indicated that teachers are more prone to insomnia compared to the general working population [39,40]. The impact of insomnia on teachers extends beyond individual well-being to affect job performance and student outcomes. Research has shown that sleep disturbances can lead to cognitive impairments, reduced creativity, and poorer decision-making, highlighting the importance of addressing sleep issues among educators for optimal performance [41].

This study explores the interconnections between teacher stress, sleep quality, and somatic symptoms during the post-COVID-19 pandemic. Research has extensively documented how the continuous demands of the teaching profession can exacerbate physical and psychological issues [42,43,44]. This contribution aims to provide insights into the nuanced ways professional and personal challenges during the pandemic impact teacher health. By examining the relationships between stress, sleep quality, and somatization disorders, the study seeks to inform policy and practice, offering guidance on supporting teacher resilience and educational efficacy amid ongoing challenges [45,46,47,48,49,50,51,52,53].

- Objectives of the Study

The primary aim of this research is to elucidate the relationships between somatization, stress levels, sleep quality, and fears related to post-COVID-19 among teachers. Specifically, the study seeks to:

- Identify and quantify the correlations between these variables.
- Develop a predictive model for somatization disorders among teachers.
- Examine how demographic and professional variables such as gender, contract type, institutional type, and years of experience influence these relationships.

- Hypotheses

Based on the outlined objectives and previous literature, the study posits several hypotheses:

- There is a significant positive correlation between the levels of stress experienced by teachers and the severity of somatic symptoms they report (in accordance with [54,55,56,57]).
- There is a significant positive correlation between poor sleep quality, stress and somatic symptoms (in accordance with [10,56,57]).
- Fears related to COVID-19 compound the effects of stress on somatic symptoms, further impairing sleep quality and overall well-being (in accordance with [58,59]).
- Demographic and professional variables significantly modulate the relationships between stress, sleep quality, and somatization (in accordance with [60]).

- Significance of the Study

The significance of this study extends beyond its empirical findings, providing critical insights into the complex interplay between psychological stress, sleep quality, and somatization among teachers in the post-COVID-19 era. By systematically identifying and quantifying the correlations between these variables, the research highlights potential pathways through which stress manifests and impacts the physical and psychological health of educators.

The research findings contribute to a deeper understanding of how fears related to COVID-19 amplify existing stressors. This knowledge is essential for educational policymakers and mental health professionals as they design and implement support systems that are sensitive to the unique challenges posed by the pandemic and its aftermath. By addressing these specific factors, interventions can be more effectively tailored to meet the current needs of teachers, enhancing their resilience and ability to cope with stress.

By bridging the gap between research and practice, this study serves as a catalyst for meaningful change, ensuring that the health of teachers is recognized as a pivotal element of educational success and sustainability in the post-pandemic world [61,62,63].

## 2. Materials and Methods

### 2.1. Participants

Participants were recruited through a voluntary process using digital ads distributed via email and social platforms used professionally by teachers. Before participating, participants were required to provide informed consent, assuring them complete confidentiality and anonymity in handling the collected data. It was also clearly explained that participation or withdrawal from the study would not impact their job position or other aspects of their professional life. Although they started the protocol compilation, 67 teachers did not finish it; therefore, they were not included in the analyses. At the end of the recruitment process that took place in April and May 2021, considering the exclusion of drop-outs, we ultimately collected 320 teachers from various educational institutions.

The gender distribution consisted of 35 males (11%) and 285 females (89%), reflecting the typical gender proportions in the teaching field. The average age of the participants was 49 years, ranging from 24 to 66 years, which allows us to explore the effects of age on sleep quality and stress levels. The participants had an average of 18 years of experience in the education field, with a standard deviation of 11 years, offering a variety of experiences and perspectives on the profession. Most participants (80%) had permanent contracts, which may affect their perception of job security and, consequently, stress levels. Following Table 1 reports the demographic and professional data of participants.

### 2.2. Procedure and Instruments

The data collection procedure was designed to ensure the integrity and confidentiality of information while adhering to ethical standards and participant privacy. Data were gathered through the electronic protocol Questbase, allowing to reach a broad sample of teachers across the Italian regions of Lazio, Molise, and Campania. Digital platforms were used to promote recruitment for the study. The platforms included popular professional networks for educators, such as LinkedIn and specific Facebook groups dedicated to teachers. Before participating, each teacher received detailed information regarding the study’s purpose, the procedures involved, potential benefits, and risks. Upon obtaining informed consent, participants were guided through the completion of an online questionnaire hosted on Questbase, a secure platform for online surveys that ensures data anonymity and security.

The questionnaire was accessible for a period of three weeks, with weekly reminders sent via email to encourage full participation. At the end of the data collection period, responses were automatically encrypted and transferred for analysis.

For a thorough assessment of the various aspects of stress, sleep quality, somatization, and fear of COVID-19, the following standardized and validated tools were used:

(1) Pittsburgh Sleep Quality Index (PSQI): This tool is used to assess the quality and patterns of participants’ sleep [64]. The PSQI consists of 19 items that add up to form seven components: subjective sleep quality, sleep latency, sleep duration, habitual sleep efficiency, sleep disturbances, use of sleeping medication, and daytime dysfunction. This instrument provides a comprehensive index of sleep quality, utilizing the Italian validation [65]. For this study, the Cronbach alpha reliability measure was 0.721.

(2) Mesure du Stress Psychologique (MSP): Developed by Lemyre, Tessier, and Fillion in 1990 [66] and validated in Italian by Di Nuovo and Rispoli in 2000 [67]. The MSP consists of 49 items that cover different aspects related to the individual’s perception of their state. This includes the cognitive-affective, physiological, and behavioral aspects, which are the three main categories that provide a global index of psychological stress. The instrument is divided into six clusters: Loss of control/irritability (Cronbach’s alpha = 0.90); Psychophysiological sensations (Cronbach’s alpha = 0.89); Sense of effort and confusion (Cronbach’s alpha = 0.87); Depressive anxiety (Cronbach’s alpha = 0.88); Physical pain and problems (Cronbach’s alpha = 0.85); Hyperactivity, Accelerations in behavior (Cronbach’s alpha = 0.86). Measurement is carried out on a four-point Likert scale, with the total score providing an index of the participant’s stress state. For this study, the global Cronbach alpha reliability measure was 0.934.

(3) Fear of COVID-19 Scale: Validated in Italian by Soraci et al., 2020 [68], this scale measures specific fear related to COVID-19 through 7 items on a Likert scale from 1 (strongly disagree) to 5 (strongly agree). It includes items such as: “I am very afraid of COVID-19”, “Thinking about COVID-19 makes me anxious”, “My hands start to sweat when I think about COVID-19”, “I am afraid of dying from COVID-19”, “When I see news and stories about COVID-19 on social media, I become nervous or anxious”, “I cannot sleep because I’m worried about getting COVID-19”, “My heart races or pounds when I think about catching COVID-19”. This scale helps determine the psychological impact of the pandemic on participants. For this study, the Cronbach alpha reliability measure was 0.888.

(4) Somatization Index (SOMAT): The index was specifically designed for this study to capture the onset or exacerbation of conditions such as high blood pressure, diabetes, high cholesterol, cardiac disorders, respiratory issues, headaches, stomach problems, skin diseases, gastrointestinal disorders, and food intolerances. The scores range from 0 (no physical issues) to 9 (greater and more numerous physical symptoms), allowing for a detailed assessment of the physical manifestations of stress among teachers. This approach was intended to provide an understanding of how stress impacts physical health, particularly in the context of the challenges posed by the COVID-19 pandemic. Participants were asked to report the onset or exacerbation in the last eight months of conditions such as high blood pressure, diabetes, high cholesterol, cardiac disorders, respiratory issues like asthma, bronchitis, etc., headaches, stomach problems (gastritis, reflux), skin diseases (erythema, psoriasis, dermatitis, urticaria), and gastrointestinal disorders (colitis, bloating, constipation), and food intolerances.

Exploratory Factor Analysis (EFA) results for the Somatization Index (SOMAT) showed a three-factor solution (Cumulative Variance Explained 57.13%): Factor 1: Metabolic Syndrome Related Symptoms with items such as High Blood Pressure, High Cholesterol, Diabetes (variance explained 23.82%); Factor 2: Gastrointestinal and Neurological Stress Responses with items such as Stomach Problems, Headaches, Intestinal Problems (Variance Explained 19.93%); Factor 3: Immune and Inflammatory Responses with items such as Cardiac Problems, Respiratory Problems, Food Intolerances, Skin Problems (Variance Explained 10.21%). 

The reliability of the SOMAT index for this study was measured with a Cronbach’s alpha of 0.705, indicating acceptable internal consistency. More specifically, the first factor’s alpha was 0.750, the second factor 0.710, and the third factor 0.668.

### 2.3. Statistical Analyses

Initially, Exploratory Factor Analysis (EFA) was conducted on the SOMAT index to identify the underlying factor structure and to ensure the validity of the instrument in capturing the dimensions of somatization among teachers. The EFA was performed using Principal Axis Factoring (PAF) with an oblique (Promax) rotation, which is appropriate given the expectation that the factors may be correlated. The number of factors to retain was determined based on multiple criteria: eigenvalues greater than 1, the scree plot, and the interpretability of the factor solutions. Items with factor loadings greater than 0.40 on a given factor were considered significant, and those with cross-loadings above 0.30 on multiple factors were carefully examined for their contribution to the scale.

Descriptive analyses were performed to provide a general overview of the sample, including mean, median, standard deviation, and frequencies for all quantitative and qualitative variables. This included demographic and professional variables such as age, gender, years of professional experience, contract type, and other relevant demographics. This step was essential in establishing a baseline understanding of the sample and ensuring the representation across different demographic and professional backgrounds.

Before proceeding with further statistical tests, the Shapiro–Wilk test was employed to assess the normality of the distribution of continuous variables. Establishing the normality of the data is crucial because it determines the appropriate statistical tests for subsequent analyses. For normally distributed variables, parametric tests can provide more powerful and precise results, whereas non-normally distributed data require non-parametric tests to avoid false conclusions.

To explore the relationships among stress, sleep quality, somatization, and fear of COVID-19, Pearson’s correlation coefficient was utilized for normally distributed variables, and Spearman’s rank correlation coefficient was used for non-normally distributed variables. These correlation analyses were instrumental in identifying and quantifying the associations between the variables, helping to highlight significant relationships that warrant further investigation.

Multiple regression analysis was conducted to evaluate the effectiveness of predictors such as stress levels, sleep quality, and somatization. This approach allowed for determining the independent impact of each predictor while controlling for other relevant variables. To ensure the validity and reliability of the results, the coefficient of determination (R^2^) was calculated, and issues of multicollinearity were assessed using the Variance Inflation Factor (VIF) and tolerance in regression analyses, normality of residuals using the Shapiro–Wilk Test and homoscedasticity using the Breusch–Pagan Test. These measures helped to confirm that the predictors in the regression models did not introduce significant redundancy, thus safeguarding the integrity of the findings. Results were presented with Beta coefficients, standard errors, t-values, and significance levels, providing a comprehensive view of how each variable contributes to the outcomes being studied.

Where necessary, independent t-tests and ANOVA (Analysis of Variance) were utilized to compare means between two groups and among more than two groups, respectively. These tests are crucial for analyzing differences based on gender, contract type, or type of educational institution, providing insights into how these factors might influence stress, sleep, and somatization differently.

To ensure the robustness of our findings, we conducted power analyses for the key statistical tests performed in this study. These analyses aimed to determine the required sample sizes to achieve a statistical power of 80% at an alpha level of 0.05 for detecting significant effects.

- Stress Levels vs. Somatization

Effect Size: 0.53; Required sample size (80% Power): 92 participants

- Sleep Quality vs. Somatization

Effect Size: 0.45; Required sample size (80% Power): 128 participants

- ANOVA (School Types vs. Somatization)

Effect Size: 2.0; Required sample size (80% Power): 15 participants per group

- ANOVA (School Types vs. Stress Levels)

Effect Size: 1.25; Required Sample Size (80% Power): 23 participants per group

- Multiple Regression

Effect Size: 0.45 (based on R-squared value of 0.31); required sample size (80% Power): 70 participants.

All analyses were performed using SPSS software (Version 26), and results were considered statistically significant at a *p*-value of less than 0.05. Power analysis was done using G*Power software (version 3.1).

## 3. Results

### 3.1. Descriptive Statistics

Our sample consisted of 320 teachers, predominantly female (89%), with ages ranging from 24 to 66 years and an average experience in teaching of approximately 18.5 years. The psychological stress levels, as measured by the Mesure du Stress Psychologique (MSP), had a mean score of 1.66, indicating a moderate level of stress across the sample. Sleep quality, measured by the PSQI, had a mean score of 7.59, suggesting that many teachers experienced poor sleep. The average score for fear of COVID-19 was 2.52 on a scale up to 5, showing a moderate level of concern among participants. Similarly, scores for anxiety and depression averaged 1.71, with physical pain also averaging the same, suggesting a commonality in the intensity of these experiences among the participants.

Table 2 below summarizes the descriptive statistics for key variables, including age, years of experience, psychological stress, anxiety and depression, and physical pain.

### 3.2. Correlation Analysis

Spearman’s correlation coefficients were calculated to determine the relationships between different psychological and physical health measures. Significant correlations were found and reported in Table 3.

Depressive anxiety was highly correlated with psychological stress (ρ = 0.84, *p* < 0.001). Psychological stress also showed a strong correlation with physical pain (ρ = 0.78, *p* < 0.001), suggesting that higher stress levels are associated with increased physical symptoms. Furthermore, the somatization index was significantly correlated with psychological stress (ρ = 0.49, *p* < 0.001), indicating that higher levels of psychological stress are associated with more pronounced somatic symptoms.

A moderate correlation was observed between anxiety and depression and hyperactivity acceleration (ρ = 0.46, *p* < 0.001). Sleep quality (PSQI) also showed moderate correlations with psychological stress (ρ = 0.54, *p* < 0.01), anxiety and depression (ρ = 0.48, *p* < 0.05), and physical pain (ρ = 0.46, *p* < 0.05), indicating that poor sleep quality is linked to higher levels of stress, anxiety, depression, and physical symptoms. Additionally, fear of COVID-19 demonstrated significant correlations with psychological stress (ρ = 0.38, *p* < 0.05), sleep quality (ρ = 0.30, *p* < 0.05), anxiety and depression (ρ = 0.39, *p* < 0.05), physical pain (ρ = 0.34), and the somatization index (ρ = 0.36, *p* < 0.001), suggesting its broad impact on both psychological and physical health factors.

### 3.3. Regression Analysis

A comprehensive regression model was developed, including the Measure of Psychological Stress (MSP), Global Pittsburgh Sleep Quality Index (PSQI), Fear of COVID-19 (FC), gender, age, and years of experience. In order to verify regression assumptions, normality of residuals, homoscedasticity, and multicollinearity have been checked. The Shapiro–Wilk test result (*p*-value = 0.062) suggests that the residuals from the regression model approximately follow a normal distribution, as the *p*-value is slightly above the 0.05 threshold, indicating a marginal acceptance of normality. This result suggests that the assumption of normality is not severely violated. The Breusch–Pagan test yields a *p*-value of 0.117, which is greater than the standard alpha level of 0.05, indicating that there is no significant evidence of heteroscedasticity in the model. This suggests that the variance of the errors is constant, satisfying one of the critical assumptions of linear regression. The VIF scores for all predictors are well below the commonly used threshold of 5, indicating that multicollinearity is not a concern in this model. This result suggests that the independent variables provide distinct information without redundancy in predicting the dependent variable.

Table 4 summarizes the comprehensive regression analysis results, including all predictors: psychological stress (MSP), sleep quality (PSQI), fear of COVID-19 (FC), gender, age, years of experience, and the interaction between age and experience. These factors were analyzed to determine their impact on psychophysical pain among teachers.

Key Findings:

- Psychological Stress (MSP): Exhibited a significant and strongly positive impact on somatic pain, suggesting that higher levels of stress are associated with increased pain (Coefficient = 1.297, *p* < 0.001). This result highlights psychological stress as a significant predictive factor for somatic pain among teachers.
- Sleep Quality (PSQI): Showed a statistically significant impact on somatic pain (Coefficient = 0.111, *p* < 0.001), indicating that poor sleep quality is linked to higher levels of somatic pain. This supports the notion that improving sleep quality may be an effective intervention to reduce somatic symptoms among teachers.
- Fear of COVID-19 (FC): Contributed significantly to the model (Coefficient = 0.368, *p* = 0.003), reflecting the current concerns affecting teachers during the pandemic. This result indicates that pandemic-related concerns can exacerbate physical symptoms associated with stress.
- Gender: Did not show a significant influence on somatic pain in this study (Coefficient = 0.285, *p* = 0.346), suggesting that the effects of stress and sleep on somatic pain do not differ significantly between genders.
- Age: Included in the model but did not show a significant effect on somatic pain (Coefficient = 0.024, *p* = 0.207), suggesting that age alone is not a significant predictor of somatic pain.
- Years of Experience: Did not significantly affect somatic pain (Coefficient = 0.010, *p* = 0.853), indicating that the length of work experience does not significantly alter the impact of stress or other factors on somatic symptoms.
- Interaction between Age and Experience: The interaction term to explore whether experience modifies the effect of age on pain did not yield significant results (Coefficient = −0.0004, *p* = 0.662), suggesting that the interaction between age and experience is not a relevant factor for somatic pain.

The regression model accounted for approximately 31.1% of the variance in somatic pain (R-squared = 0.311), indicating substantial explanatory power. The adjusted R-squared value of 0.296 indicates that approximately 29.6% of the variance in the dependent variable can be explained by the independent variables included in the model. This suggests a moderate explanatory power of the model, considering the number of predictors used. Independent *t*-tests were used to explore differences in psychophysical pain based on gender, showing no significant disparities (*p* > 0.05). ANOVA was conducted to examine differences in psychophysical pain across different types of contracts and school types, which did not yield significant variations (*p* > 0.05).

#### Analysis of Hypotheses

The present study examined several hypotheses concerning the relationships between stress levels, sleep quality, and somatic symptoms among teachers, as well as the impact of COVID-19-related fears and demographic variables.

To test Hypothesis 1, which posited a significant positive correlation between the levels of stress experienced by teachers and the severity of somatic symptoms, we performed a Pearson correlation analysis. The results showed a significant positive correlation (r = 0.49, *p* < 0.001), confirming that higher levels of stress are associated with more severe somatic symptoms.

Hypothesis 2, which suggested a significant positive correlation between poor sleep quality, stress, and somatic symptoms, was examined by analyzing the correlations between sleep quality (PSQI), stress (MSP), and somatic symptoms. Significant positive correlations were found between sleep quality and stress (r = 0.54, *p* < 0.01), and between sleep quality and somatic symptoms (r = 0.44, *p* < 0.001). These findings support the hypothesis that poor sleep quality exacerbates stress and is linked to increased somatic symptoms.

To evaluate Hypothesis 3, which proposed that fears related to COVID-19 compound the effects of stress on somatic symptoms, we used multiple regression analysis incorporating the Fear of COVID-19 scale as an additional predictor. The analysis revealed that COVID-19-related fears significantly contributed to the model, with a beta coefficient of 0.368 (*p* = 0.003), indicating that fears related to the pandemic exacerbate the effects of stress on somatic symptoms and impair sleep quality.

Hypothesis 4, which suggested that demographic and professional variables significantly modulate the relationships between stress, sleep quality, and somatization, was assessed using ANOVA tests. The results did not show significant differences across different school types, indicating that these variables do not modulate the relationships between the studied factors.

## 4. Discussion

This study’s findings focus on the significant interplay between psychological stress, sleep quality, and somatic symptoms among teachers in the post-COVID-19 era, enriching our understanding of how these factors coalesce to impact educator well-being and professional efficacy. Notably, the role of pain, particularly somatic pain related to stress and poor sleep, is a critical aspect of findings that merits deeper examination within the context of existing research and novel insights provided by this study.

Our research extends previous findings by explicitly focusing on somatic pain as a manifestation of psychological stress exacerbated by disrupted sleep patterns [69,70,71]. This aligns with and expands upon psychosomatic theory, which posits that emotional and psychological stressors can manifest physically, thereby affecting overall health and functioning. Neuroscientific research indicates that chronic stress can trigger biological pathways that lead to the development of physical symptoms, such as headaches, muscle tension, gastrointestinal problems, and fatigue [72,73,74,75]. These symptoms are not just byproducts of stress but are direct manifestations of the body’s response to prolonged exposure to elevated cortisol levels and sympathetic nervous system activity [76,77,78]. Such a relationship is especially pronounced under conditions like those created by the COVID-19 pandemic, which significantly elevated stress levels among teachers due to abrupt transitions to online teaching, heightened concerns over personal and student health, and the challenges of maintaining educational standards amidst global uncertainty [79,80,81,82,83].

Comparative analyses with pre-existing studies, such as those by Fontana et al. [10] and Cropley et al. [16], reveal that while the correlation between stress and physical health symptoms like pain is well-established, our study contributes novel insights by highlighting the role of sleep quality. These findings suggest that interventions aimed at improving sleep quality could be particularly effective in alleviating pain symptoms associated with stress, offering a targeted approach to enhancing teacher well-being.

While our findings did not establish sleep quality as a mediator in the relationship between psychological stress and somatic symptoms, its significant association with both constructs emphasizes its critical role in overall health management. Poor sleep quality can exacerbate both psychological and physical health issues, creating a feedback loop that can severely impact an individual’s health and quality of life. This relationship is particularly concerning in professions characterized by high stress, such as teaching, where the demands of the job can frequently lead to disrupted sleep patterns [84,85].

Research by O’Connor et al. [86] supported the notion that inadequate sleep can amplify the subjective experience of stress. When teachers suffer from poor sleep quality, their ability to manage day-to-day stressors effectively is compromised. This not only worsens their perception of stress but may also lead to a heightened experience of somatic symptoms such as muscle tension, headaches, and fatigue. These symptoms can further disrupt sleep, perpetuating a cycle of stress and poor health that can be challenging to break [87,88,89].

In integrating our findings with the broader literature, it is evident that while many studies have focused on the psychological impacts of stress, few have explored the complex relationship between stress, sleep, and physical pain in the wake of a global crisis. Our study bridges this gap by providing empirical evidence of these dynamics and suggesting practical interventions that can be implemented within educational settings.

The vulnerability of teachers to stressors, exacerbated during such crisis conditions, confirms the need for targeted support systems within educational environments. Programs designed to reduce stress and manage somatic symptoms among teachers could not only improve their health outcomes but also enhance their ability to fulfill their roles effectively [90,91,92,93,94,95].

The unique contributions of this study are highlighted by its timing and context, which was conducted during the post-pandemic period when teachers were navigating the return to traditional teaching paradigms or hybrid models. This transitional phase presented unique stressors that are not typically covered in the literature focused on the immediate impacts of the pandemic. By addressing these evolving challenges, our study provides timely and contextually relevant insights into the ongoing adjustments and stressors faced by educators and the resultant physical manifestations such as pain.

By comparing our results with studies conducted in other high-stress professions, we can discern that the mechanisms linking stress, sleep, and pain are universally applicable yet exhibit unique characteristics in educational settings due to the specific nature of teaching responsibilities and stressors. Consistent with prior studies, such as those by Fitchett et al. [96] and McCarthy et al. [97], which highlighted the susceptibility of educators to occupational stress, this study adds to the emerging body of evidence that the educational sector must prioritize teacher health as a critical aspect of educational quality. These studies collectively underscore the necessity of adopting holistic approaches to health in educational policy and practice, recognizing the interconnected nature of psychological and physical well-being [98,99,100,101,102,103].

## 5. Limitations and Future Directions

This study provides critical insights into the complex interplay between psychological stress, sleep quality, and somatic symptoms among teachers in the post-COVID-19 era. While the findings enrich our understanding of these factors, it is crucial to acknowledge certain limitations that might influence the interpretation and generalizability of the results. The recruitment process was voluntary, which could introduce self-selection bias. Participants who volunteered may differ significantly from those who did not, potentially skewing the results towards individuals with greater health consciousness or higher perceived stress. The demographic and geographical representation of the sample was confined to specific regions, which may limit the broader applicability of the findings to other educational contexts or cultural settings. The study’s results may also be influenced by response bias, with participants potentially underreporting certain symptoms due to social desirability. Researcher bias could also influence the interpretation of data, where preconceived hypotheses might subtly shape the analysis. Addressing these limitations in future research is critical. Employing randomized sampling methods can enhance representativeness and reduce selection bias. Longitudinal studies would provide stronger evidence of causality between teacher stress and health outcomes. Refining existing measurement tools or developing new instruments tailored to the post-pandemic context will enhance the precision and relevance of research findings.

Integrating physiological measures, such as cortisol levels for stress and actigraphy for sleep quality, could enhance the objectivity of the data. These methods would help validate self-reported measures and reduce bias, providing a more accurate assessment of the physiological underpinnings of stress and sleep disturbances.

Despite these limitations, the study significantly contributes to the literature by highlighting the significant role of sleep quality in the relationship between stress and somatic symptoms. This suggests that interventions aimed at improving sleep could be particularly effective in alleviating symptoms associated with stress, offering a targeted approach to enhance teacher well-being. Comparing these results with findings from other high-stress professions reveals that while the mechanisms linking stress, sleep, and pain are universally applicable, they exhibit unique characteristics in educational settings due to specific stressors related to teaching responsibilities.

## 6. Conclusions

This study has advanced our understanding of the interrelationships among psychological stress, sleep quality, and somatization among teachers in the post-COVID-19 era. It highlights the critical role of sleep quality in managing stress-induced somatic symptoms and the heightened impact of the pandemic on teachers’ mental and physical health. Findings suggest that improved sleep quality can mitigate the adverse effects of stress on physical health, thereby enhancing overall teacher well-being. The unique conditions of the post-pandemic teaching environment, characterized by the rapid shift to digital teaching platforms and persistent uncertainties, have intensified these challenges, underscoring the need for targeted interventions.

Educational policymakers should consider integrating structured support systems within schools to assist teachers in managing stress more effectively. Recommended initiatives include training on sleep hygiene, stress management programs, and access to mental health support. Such interventions are essential for improving teachers’ quality of life and their ability to foster positive educational outcomes. Further investigation is needed to track these phenomena over time through longitudinal studies. This approach would help clarify the long-term impacts of stress and poor sleep on somatization among teachers. Expanding the scope of research to include more diverse geographic and demographic contexts could help verify the generalizability of these findings. Future studies should aim for more randomized sampling methods to enhance representativeness. Moreover, updating the measurement tools used to assess stress and sleep quality specifically tailored for post-pandemic educational settings could provide more precise data.

## Figures and Tables

**Table 1:** Data of the participants.

Characteristic	Value
Total Participants	320
Male	35 (10.94%)
Female	285 (89.06%)
Average Age	48.87 years
Age Range	24 to 66 years
Age Standard Deviation	9.33 years
Average Years of Experience	18.48 years
Years of Experience Range	1 to 42 years
Experience Standard Deviation	10.65 years
Permanent Contracts	256 (80.00%)
Temporary Contracts	64 (20.00%)
Preschool	25 (7.81%)
Elementary School	139 (43.44%)
Middle School	30 (9.38%)
High School	126 (39.38%)

**Table 2:** Summary Statistics of Study Variables.

Variable	Mean	Std Dev	Min	Max
Psychological Stress (MSP)	1.66	0.44	1	3.17
Sleep Quality (PSQI)	7.59	3.80	1	21
Fear of COVID-19 (FC)	2.52	0.83	1	5
Depressive Anxiety (DA)	1.70	0.66	1	4
Physical Pain (PP)	1.71	0.67	1	4
Hyperactivity and Acceleration (HA)	1.89	0.64	1	4
Years of Experience (YE)	18.47	10.65	1	42
Somatization Index	2.45	2.89	0	9

**Table 3:** Correlation Coefficients among Study Variables.

Variables	MSP	PSQI	FC	DA	PP	HA	YE	SO
Psychological Stress (MSP)	1.00							
Sleep Quality (PSQI)	0.54 **	1.00						
Fear of COVID-19 (FC)	0.38 *	0.30 *	1.00					
Depressive Anxiety (DA)	0.84 ***	0.48 *	0.39 *	1.00				
Physical Pain (PP)	0.78 ***	0.46 *	0.34	0.60 **	1.00			
Hyperactivity and Acceleration (HA)	0.70 ***	0.32	0.21	0.46 *	0.36	1.00		
Years of Experience (YE)	−0.04	0.03	0.01	0.05	−0.02	−0.02	1.00	
Somatization Index	0.49 ***	0.44 ***	0.36 ***	0.39 ***	0.53 ***	0.27 ***	−0.00	1.00

* indicates *p* < 0.05; ** indicates *p* < 0.01; *** indicates *p* < 0.001.

**Table 4:** Impact of Psychological Factors and Demographics on Psychophysical Pain Among Teachers.

Variable	Beta Coefficient	Std. Error	t-Value	*p*-Value	95% CI
Intercept	−2.929	1.034	−2.833	0.005	−4.963, −0.895
Psychological Stress (MSP)	1.297	0.258	5.020	<0.001	0.789, 1.805
Sleep Quality (PSQI)	0.111	0.029	3.766	<0.001	0.053, 0.169
Fear of COVID-19 (FC)	0.368	0.122	3.010	0.003	0.127, 0.609
Gender	0.285	0.301	0.945	0.346	−0.308, 0.878
Age	0.024	0.019	1.265	0.207	−0.013, 0.061
Years of Experience	0.010	0.053	0.186	0.853	−0.094, 0.114
Age x Experience	−0.0004	0.001	−0.437	0.662	−0.002, 0.001

## Data Availability

The raw data supporting the conclusions of this article will be made available by the corresponding author upon request. The data are not publicly available due to specific ethical and privacy considerations.
